# Impact of multivessel coronary artery disease on reperfusion success in patients with ST-elevation myocardial infarction - insights from cardiac magnetic resonance imaging

**DOI:** 10.1186/1532-429X-17-S1-Q48

**Published:** 2015-02-03

**Authors:** Suzanne de Waha, Ingo Eitel, Steffen Desch, Georg Fuernau, Philipp Lurz, Gerhard Schuler, Holger Thiele

**Affiliations:** 1 Heart Center Leipzig, Leipzig, Germany; 2 Heart Center Bad Segeberg, Bad Segeberg, Germany; 3 Heart Center Luebeck, Luebeck, Germany

## Background

A significant portion of patients with ST-elevation myocardial infarction (STEMI) display multivessel coronary artery disease (MVD). However, data on the association of MVD and reperfusion success are scarce. Thus, we thought to analyse the impact of MVD on infarct size, microvascular obstruction (MO), and myocardial salvage index (MSI) assessed by cardiac magnetic resonance imaging (CMR) in a large unselected cohort of patients with STEMI reperfused by primary percutaneous coronary intervention (PCI).

## Methods

STEMI patients reperfused by primary PCI (n=1074) within 12 hours after symptom onset underwent CMR 3 days after the index event (interquartile range [IQR] 2-4). Infarct size and MO were measured 15 min after gadolinium injection. T2-weighted and contrast-enhanced CMR were then used to calculate MSI. Severity of coronary artery disease was graded as single-vessel disease compared to MVD. Further, a detailed set of clinical, angiographic, and electrocardiographic parameters was recorded. The primary endpoint was defined as a composite of death, non-fatal myocardial reinfarction, and congestive heart failure (MACE). Clinical follow-up was conducted after 12 months.

## Results

MVD was present in 48.5% (n=521) of all patients. Patients with MVD were older (66 [IQR 55-73] vs. 60 [IQR 50-70] years, p<0.001) and more often diabetics (26.3 vs. 17.5%, p=0.001) in comparison to those with single-vessel disease. Angiographic reperfusion success defined as TIMI-flow III post-PCI (87.6 vs. 88.1%, p=0.92), and ST-segment resolution (60 [IQR 25;80] vs. 60 [30;80]%, p=0.18) were similar between both groups.

Patients with MVD displayed no significant differences in infarct size (17.5 [IQR 8.4-26.4] vs. 16.0 [IQR 8.5-24.4]%LV, p=0.15) and extent of MO (0.4 [IQR 0.0;1.6] vs. 0.3 [IQR 0.00-1.7]%LV, p=0.71), as well as MSI (52 [IQR 33-74] vs. 53 [IQR 36-72], p=0.48) in comparison to patients with single-vessel disease.

Finally, the presence of MVD was significantly associated with the time-dependent occurrence of MACE (log-rank comparison p=0.004).

## Conclusions

MVD is not associated with impaired reperfusion success assessed by CMR. The adverse clinical outcome of patients with MVD might thus rather be explained by more advanced coronary artery disease itself and unfavourable baseline characteristics.

## Funding

None.

